# Minor tumor infiltrating B cells opened a door to reveal and eliminate cancer initiating cells in metastatic melanomas

**DOI:** 10.1186/2051-1426-2-S1-P4

**Published:** 2014-02-24

**Authors:** Beatrix Kotlan, Gabriella Liszkay, Gyorgy Naszados, Judit Olasz, Szabolcs Horvath, Vanda Plotar, Andras Szollar, Istvan Nagy Vamosi, Akos Savolt, Laszlo Toth, Orsolya Csuka, Maria Godeny, Miklos Kasler, Francesco M Marincola

**Affiliations:** 1 Molecular Immunology and Toxicology, National Institute of Oncology, Budapest, Hungary; 2 Oncodermatology, National Institute of Oncology, Budapest, Hungary; 3 Radiological Diagnostics, National Institute of Oncology, Budapest, Hungary; 4 Pathogenetics, National Institute of Oncology, Budapest, Hungary; 5 Surgical and Molecular Tumorpathology, National Institute of Oncology, Budapest, Hungary; 6 Oncosurgery, National Institute of Oncology, Budapest, Hungary; 7 Board of Directors, National Institute of Oncology, Budapest, Hungary; 8 Research, Sidra Medical and Research Center, Doha, Qatar

## Background

The theory and investigations on cancer stem cells (CSCs) have received growing attention, as these cells are responsible for the failure of cancer therapeutic strategies and the return of cancer.

## Materials and methods

A complex tumorimmunological study on primary and metastatic cancerous tissue biopsies and peripheral blood of patients with malignant melanomas (n = 153) has been performed with ethical permission (ETT TUKEB 16462- 02/2010) and patients’ formal consent.

## Results

We developed a methodology to select cancer initiating cells from fresh cancer cell cultures of malignant melanomas. Characteristic cell growth pattern, spheroid forming, CSC markers, like CD133, Nestin, ABCB5, CD20 and unique GD3 ganglioside expression were defined by immunofluorescence assay. Additionally tumor infiltrating B cells’ sialilated glycosphingolipid binding was defined by antibody phage display and immunglobulin repertoire analysis. Real Time PCR gene expression studies were done to reveal molecular parameters of regulatory mechanisms.

## Conclusion

We provide here a novel strategy to detect cancer initiating cells in metastatic melanomas by double labeling with anti CD20 antibodies and sialilated glycosphingolipid antigen specific immunglobulins. With antibody engineering present findings might be turned into a novel cancer therapeutic approach targeting cancer stem cells.

